# Diaphragmatic ultrasound and thoracic fluid content for prediction of non-invasive ventilation failure in neonates: a randomized controlled trial

**DOI:** 10.1007/s00431-025-06605-8

**Published:** 2025-12-09

**Authors:** Essam Mahmoud Elgendy, Heba Saied Elmahdy, Mohamed Abd Elatif Nassar, Lamiaa Khaled Zidan, Mohamed Adel Eltomey, Hamed Mohamed Elsharkawy

**Affiliations:** 1 Pediatric Department, Faculty of Medicine, Tanta University, Gharbia Governorate, El Bahr St., Tanta Qism2, Tanta, 31527 Egypt; 2 Radiology Department, Faculty of Medicine, Tanta University, Tanta, Egypt

**Keywords:** Lung ultrasound, Thoracic fluid content, Prediction, Non-invasive ventilation failure, Neonates

## Abstract

Non-invasive ventilation (NIV) remains the standard of care for preterm infants with respiratory distress syndrome (RDS); however, NIV failure is frequent and often linked to adverse outcomes. This study evaluated diaphragmatic ultrasound and thoracic fluid content (TFC) as early predictors of NIV failure in preterm neonates. In this prospective randomized controlled trial, 90 preterm neonates (28–34 gestational weeks) with RDS requiring NIV as initial respiratory support were randomized into three groups: (1) nasal continuous positive airway pressure (nCPAP), (2) nasal intermittent positive pressure ventilation (NIPPV), and (3) nasal high-frequency oscillatory ventilation (NHFOV). Diaphragmatic thickening fraction (DTF) and excursion (DE) were measured by ultrasound, while TFC was assessed using electrical cardiometry (EC). Measurements were obtained within the first 3 h of life and repeated either at 24 h in successful cases or immediately before intubation in failed cases. DTF and DE were significantly higher in neonates with successful NIV compared with those who failed (all *p* < 0.001), while TFC was significantly higher in failed cases within NIPPV and NHFOV groups (*p* < 0.001). DTF and DE showed excellent predictive accuracy (AUC 0.90 and 0.89), outperforming TFC (AUC 0.81, *p* < 0.01). Both DTF (adjusted OR = 0.89, 95% CI 0.83–0.96) and DE (adjusted OR = 0.38, 95% CI 0.20–0.73) independently predicted NIV failure. The combined DTF–TFC model provided the highest discrimination (AUC 0.93, 95% CI 0.89–0.97).

*Conclusions*: Diaphragmatic ultrasound and TFC-derived EC represent simple, non-invasive, and reliable tools for predicting NIV failure in preterms with RDS. Clinical trial registration: clinicaltrials.gov/ NCT07148102; registered August 22, 2025.

**Table:** 

**What is Known:** • *NIV is widely used in preterm neonates with RDS, but predicting NIV failure remains challenging. Lung ultrasound score and thoracic fluid content have been explored as bedside predictors of NIV outcomes, with variable accuracy.*. • *Diaphragmatic ultrasound parameters (DTF and DE) have shown potential in assessing respiratory function in neonates*.
**What is New:** • *DTF and DE measured by ultrasound show a strong correlation with TFC obtained by EC in predicting NIV failure. Additionally, DTF and DE have higher predictive accuracy than TFC in identifying preterm neonates at risk of NIV failure*. • *Early assessment of DTF and DE may serve as a valuable bedside tool for monitoring lung function during early application of NIV in preterm infants*.

**Supplementary Information:**

The online version contains supplementary material available at 10.1007/s00431-025-06605-8.

## Introduction

Respiratory distress syndrome (RDS) remains a leading cause of mortality and morbidity among preterm infants. While essential in RDS management, invasive mechanical ventilation (IMV) can cause complications, such as ventilator-induced lung injury, infections, and the development of bronchopulmonary dysplasia (BPD) [1–3].

Noninvasive ventilation (NIV) provides several modes of respiratory support to maintain spontaneous breathing without the need for endotracheal intubation so it is widely used as a corner treatment strategy in preterm infants with RDS. Commonly used modalities are nasal continuous positive airway pressure, nasal intermittent positive pressure ventilation, heated high-flow nasal cannula, and nasal high-frequency oscillatory ventilation [4].

The key clinical benefit of the early initiation of NIV is the avoidance of IMV with all the related lung injury and long-term complications. However, its efficacy may vary, and the success rate largely depends on the gestational age [5].

Notably, infants who fail NIV have an increased risk of death, pneumothorax, and BPD, among other morbidities. Clinical indicators of NIV failure include frequent apnea, a high oxygen requirement, and hemodynamic instability [6].

Neonatal point-of-care ultrasound (POCUS) applications have increased over the past decade in emergent situations, differentiating neonatal respiratory pathologies and predicting neonatal morbidity [7].

The diaphragm is the main muscle of respiration, which undertakes most of the work of breathing in neonates. Diaphragmatic function can be evaluated using several modalities, including electromyography, transdiaphragmatic pressure measurements via esophageal and gastric pressure transducers, phrenic nerve stimulation, fluoroscopy, and analysis of ventilator-generated pressure waveforms. While these techniques offer valuable insights, their application in the neonatal population is often limited by the need for specialized equipment, technical expertise, and complex interpretation. POCUS is an easy, non-invasive, and bedside tool to assess diaphragmatic structure and function, including diaphragmatic thickening fraction (DTF) and excursion (DE) [8–10].

Nowadays, there is an increasing interest in cardiac factors, such as lung congestion and hypervolemia, as contributing elements in NIV failure. Electrical cardiometry-derived thoracic fluid content (EC-derived TFC) is measured using impedance cardiography technology to represent the whole (extravascular, intravascular, and intrapleural) fluid component in the thorax [11, 12], and it showed a strong correlation with lung ultrasound in assessing extravascular lung water [13].

This study hypothesizes that **diaphragmatic assessment by POCUS and EC-derived TFC could be used as early predictive tools for identifying NIV failure in premature neonates with RDS.**

## Methods

### Study design, setting, and ethical approval

This prospective randomized controlled study was conducted at the NICU, Tanta University Hospitals, Egypt. After the Research Ethics Committee of Tanta University Hospitals approved the study (approval code: 34,645/4/21), and written informed consent was obtained from the parents of all participating neonates, the study was conducted in accordance with the Declaration of Helsinki.

### Study period and population

**Fig. 1:**
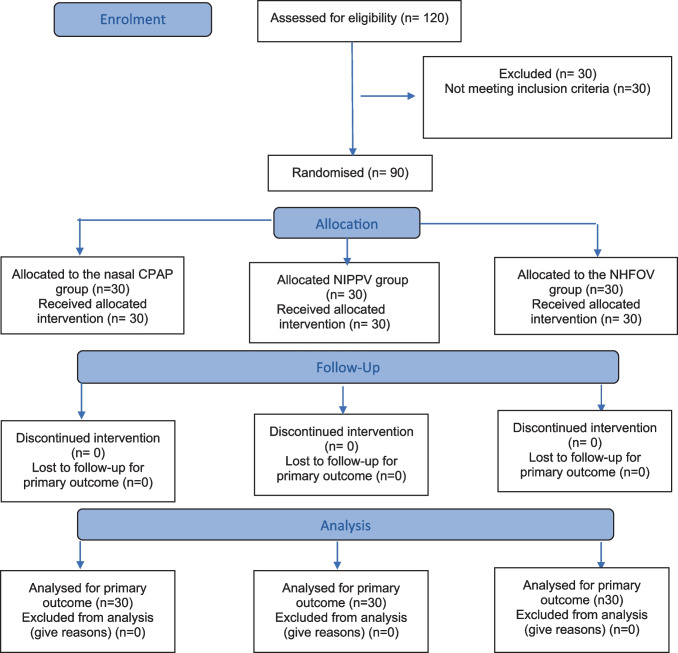
CONSORT flow diagram

Patients were enrolled from May 2021 to May 2023. During the research period, a total of 120 preterm neonates who were admitted to the NICU with respiratory distress syndrome were assessed for eligibility. Thirty neonates were excluded based on the exclusion criteria, while 90 neonates were enrolled and prospectively followed up for NIV failure or success (Fig. 1).

### Inclusion criteria included

- GA 28–34 weeks
- Diagnosis of respiratory distress syndrome required NIV as the initial mode of respiratory support within 30 min of birth.

*Exclusion criteria* included intubation in the delivery room, complex congenital heart disease, major congenital anomalies in the lungs or airways, and other causes of respiratory distress unrelated to RDS.

### Randomization and group allocation

The preterm neonates were randomly allocated in a 1:1:1 ratio to one of three groups using a simple randomization procedure (computer-generated random numbers). The allocation was done using numbered, sealed envelopes, which were only opened when the random allocation was performed. This procedure was done by a research coordinator who was not involved in recruitment, assessment, or study interventions. Ninety neonates were equally divided into three treatment groups (30 patients each): (1) nasal continuous positive airway pressure (nCPAP), (2) nasal intermittent positive pressure ventilation (NIPPV), and (3) nasal high-frequency oscillatory ventilation (NHFOV). Twins were assigned to the same treatment group, and infants enrolled in a specific group were not allowed to switch to another group during the study.

### Intervention

All infants were transferred to the NICU within 30 min of birth and received assigned NIV support. Surfactant and/or caffeine citrate were administered according to the international guidelines and the local clinical practice. Poractant alfa (Curosurf) 200 mg/kg was given as a rescue surfactant to treat worsening babies with RDS when FiO2 > 0.30 to maintain a target saturation range of 90–94%, as recommended by the European RDS guidelines 2022 [17], by the INSURE (intubation, surfactant, extubation) technique. Caffeine citrate was given to infants experiencing apnea, with an initial loading dose of 20 mg/kg followed by a maintenance dose of 5–10 mg/kg per day [17]. Infants remained on the assigned NIV mode until weaning or intubation. Those extubated within 72 h were classified as NIV failures. After extubation, they were returned to the same NIV mode to which they had originally been assigned, ensuring adherence to the randomization protocol and preventing intergroup cross-over.

### Study definitions

NIV failure was defined as requiring invasive ventilation within the first 72 h of life based on the following criteria for invasive ventilation: Frequent apnea (≥ 4 episodes/h), or apnea not responding to stimulation (need bag-mask ventilation ≥ 2 times/h), excessive breathing effort despite noninvasive respiratory support (Downes score ≥ 7) [14], hypercarbia PaCO_2_ ≥ 65 mmHg, and low blood oxygen tension (PaO_2_ < 50 mm Hg or FiO_2_ > 0.6 to maintain SpO_2_) [15, 16].

### Study outcomes

The primary outcome was to assess the predictive value of DTF, DE, and TFC for NIV failure in preterm neonates with RDS while secondary outcomes included comparing success rates among CPAP, NIPPV, and NHFOV groups, as well as evaluating correlations between DTF, DE, and TFC.

### Data gathering

- Clinical and laboratory assessment: A detailed history and comprehensive clinical examination were conducted for all enrolled infants. Laboratory tests included a complete blood count (CBC), C-reactive protein (CRP), assessments of liver and kidney function, capillary blood gases (CBG), random blood glucose levels, and serum electrolytes. Moreover, chest X-rays, echocardiography, and transcranial ultrasound were performed as necessary.
- Diaphragmatic ultrasound: Ultrasonographic examinations were performed by using Siemens Acuson X300 ultrasound system (electronic caliper resolution is 0.01 mm) equipped with 10 to 5 MHz transducer (Siemens Health Care GmbH, Erlangen, Germany) within the first 3 h of birth (1st measurement) and repeated either at 24 h in cases with successful NIV or immediately before intubation when NIV failed (2nd measurement). The evaluations were always performed by a single trained and experienced operator, with an assistant providing containment and comfort to the participants who were positioned in a supine position with their heads in the midline. The transducer was directed cranially, dorsally, and medially over the right subcostal region between the anterior axillary and the midclavicular lines to keep the radial beam perpendicular to the posterior third of the right hemidiaphragm. The right hemidiaphragm was chosen for all participants due to better feasibility and reproducibility. B-mode ultrasound optimized the image of the diaphragm as a three-layer structure of a central non-echogenic layer and two echogenic layers (peritoneum and pleura). Subsequently, the M-mode ultrasonography was screened, and the image was frozen after ensuring regular up and down movement of the diaphragmatic line that reflects regular breathing. The perpendicular distance between the most caudal point of this line during inspiration and the most caudal point during expiration represents the DE. The thickness of the diaphragmatic line at end-inspiration (Tdi insp) (upward slope) and end-expiration (Tdi exp) (downward slope) represented the diaphragmatic inspiratory and expiratory thickness, respectively. DTF was calculated using the formula: DTF = (Tdi insp – Tdi exp/Tdi exp) × 100. The technique and measurements were repeated up to three respiratory cycles, and the average value was recorded. Avoidance of diaphragmatic measurements during an infant’s crying or sighing movement was considered [18, 19].
- Thoracic fluid content measurement: TFC was assessed using a portable noninvasive electrical cardiometer (ICON, Osypka Medical, Berlin, Germany) by a single operator at the time points of diaphragmatic ultrasound (1st and 2nd measurements). Four skin electrodes were placed on the forehead, the left cervical region, the left mid-axillary area near the xiphoid process, and the left thigh to calculate TFC as the inverse of thoracic base impedance (1/base impedance). TFC serves as a key parameter for detecting pulmonary fluid overload and tissue edema. Data collection was performed continuously over 30 s, with the final readings determined by averaging the highest and lowest recorded values.

### Sample size calculation

Sample size calculation was based on the area under the receiver-operating characteristic (ROC) curve (AUC). Based on previous neonatal data [24], a conservative target AUC of 0.84 was adopted for calculation, with a null AUC of 0.50, *α* = 0.05 (two‐sided), power = 0.80, and an expected success‐to‐failure ratio of approximately 2.1:1 (failure prevalence 32–35%). The minimum total sample required to demonstrate an AUC significantly greater than 0.5 was about 55 neonates. To ensure adequate precision for secondary analyses, including comparisons across the three NIV modalities (nCPAP, NIPPV, and NHFOV; 1:1:1 allocation) and to maintain 95% confidence interval widths of ± 10% for sensitivity and specificity estimates, the sample was increased to 30 infants per group (total *n* = 90).

### Statistical analysis

Data analysis was conducted using SPSS software version 27 (IBM©, Chicago, IL, USA). The normality of data distribution was assessed using the Shapiro–Wilk test and by visual inspection of histograms. Parametric continuous variables are presented as mean ± standard deviation (SD) and were compared among groups using one-way analysis of variance (ANOVA), followed by Tukey’s post hoc test for pairwise comparisons. Non-parametric continuous variables are expressed as median (interquartile range, IQR) and were analyzed using the Kruskal–Wallis test, with Mann–Whitney U tests for post hoc pairwise analyses. Categorical variables are summarized as counts and percentages and compared using the chi-square or Fisher’s exact test, as appropriate. In cases where the contingency tables contained more than two categories and one or more expected cell counts were less than five, an exact probability test was performed using the Monte Carlo simulation method (10,000 replicates) to estimate *p*-values with higher accuracy. Correlations between quantitative parameters (DTF, DE, and TFC) were assessed using Pearson’s correlation coefficient for normally distributed variables and Spearman’s rho for non-normal data. Diagnostic performance of DTF, DE, and TFC in predicting NIV failure was evaluated by ROC curves and calculating the AUC, sensitivity, specificity, positive predictive value (PPV), and negative predictive value (NPV). Differences between AUCs were compared using DeLong’s test using MedCalc software v15.0. To account for potential confounding by maturity, multivariable logistic regression including GA, birth weight (BW), and either DE or DTF was performed to evaluate independence of associations. Partial correlations adjusting for GA and BW were also explored. Model calibration (Hosmer–Lemeshow), discrimination, and explained variance (Nagelkerke *R*^2^) were reported. Values of *p* < 0.05 were considered statistically significant. No interim analyses or predefined stopping rules were planned for this trial. The study was conducted according to the original protocol and completed as scheduled.

## Results

Baseline demographic and clinical characteristics across the three groups were comparable, and there was no significant difference between any parameter (*p* > 0.05), except for mode of delivery, with cesarean section being statistically higher in the CPAP group compared to NIPPV and NHFOV (*p* = 0.007) (Table 1).

**Table 1:** Baseline demographic and clinical characteristics of the study groups

	CPAP (*n* = 30)	NIPPV (*n* = 30)	NHFOV (*n* = 30)	*p*
Gestational age (weeks); mean ± SD	30.1 ± 2.0	30.3 ± 2.3	30.2 ± 2.0	0.886
Male sex; *n* (%)	19 (63.3%)	15 (50.0%)	15 (50.0%)	0.488
CS delivery mode; *n* (%)	29 (96.7%)	20 (66.7%)	20 (66.7%)	0.007^*^
Weight (g); mean ± SD	1820 ± 590	1780 ± 440	1820 ± 590	0.529
Antenatal steroids; *n* (%)	29 (96.7%)	27 (90.0%)	28 (93.3%)	^MC^*p* = 0.856
PROM; *n* (%)	5 (16.7%)	4 (13.3%)	7 (23.3%)	0.587
APGAR (5th min); median (IQR)	9.0 (7.0–9.0)	9.0 (7.0–9.0)	9.0 (7.0–9.0)	0.081
CRIB-II Score; mean ± SD	6.8 ± 1.5	7.0 ± 1.4	7.2 ± 1.6	0.482
Postnatal age at enrollment (*h*); mean ± SD	6.2 ± 2.4	6.5 ± 2.1	6.4 ± 2.3	0.814
RDS grading by X-ray; median (IQR)	3 (2.0–3.0)	3 (2.0–3.0)	3 (2.0–3.0)	0.160
Duration of NIV (days); mean ± SD	6.76 ± 4.27	7.95 ± 3.22	4.0 ± 2.57	0.002^*^
	*p*_1_ = 0.546, ***p***_**2**_ = 0.038^*^, *p*_**3**_ = 0.001^*^			

*GA* gestational age, *SD* standard deviation, *n* number, *C.S.* cesarean section, *IQR* interquartile interval (25–75th percentiles), *p*-value for comparing the studied groups, *MC* Monte Carlo, *CPAP* continuous positive airway pressure, *NIPPV* noninvasive positive pressure ventilation, *NHFOV* nasal high frequency oscillatory ventilation, *PROM* premature rupture of membrane, *CRIB-II* Clinical Risk Index for Babies II, *RDS* respiratory distress syndrome

^*^Significant *p*-value < 0.05

Regarding outcomes, the success rate of NIV did not differ significantly between the study groups (CPAP 50.0%, NIPPV 63.3%, NHFOV 66.7%; *p* = 0.378). Additionally, compared with CPAP, the odds of NIV success were insignificantly higher with NIPPV (OR 1.73, 95% CI 0.62–4.84) and NHFOV (OR 2.00, 95% CI 0.70–5.68). However, the duration of NIV was significantly shorter in the NHFOV group (4.0 ± 2.57 days) compared with NIPPV (7.95 ± 3.22 days, *p* = 0.001) and CPAP (6.76 ± 4.27 days, *p* = 0.038) (Tables 1 and 2).

**Table 2:** NIV outcomes across the study groups

	CPAP; (*n* = 30)	NIPPV; (*n* = 30)	NHFOV; (*n* = 30)	*p*	OR (95% CI)
**NIV Outcome** **Success rate *n* (%)**	15 (50.0%)	19 (63.3%)	20 (66.7%)	0.378	**NIPPV vs CPAP:** 1.73 (0.62–4.84) **NHFOV vs CPAP:** 2.00 (0.70–5.68) **NHFOV vs NIPPV:** 1.16 (0.40–3.35)

*n* number, *p*-value for comparing the studied groups, *CPAP* continuous positive airway pressure, *NIPPV* noninvasive positive pressure ventilation, *NHFOV* nasal high frequency oscillatory ventilation, *OR* odds ratio, *CI* confidence interval

To address potential confounding by maturity, baseline characteristics were comparable between success (*n* = 54) and failure (*n* = 36) groups, including GA (30.3 ± 2.1 vs 30.1 ± 2.2 weeks) and BW (1800 ± 520 vs 1830 ± 560 g), with no significant differences (Table 3).

**Table 3:** Baseline demographic and clinical characteristics of the study neonates according to NIV outcome

	Success group; (*n* = 54)	Failure group; (*n* = 36)	*p*-value	MD or OR (95% CI)
Gestational age (weeks); mean ± SD	30.3 ± 2.1	30.1 ± 2.2	0.640	+ 0.20 (− 0.68–1.08)
Birth weight (g); mean ± SD	1800 ± 520	1830 ± 560	0.750	− 30 (− 260–200)
Male sex, *n* (%)	28 (51.9%)	21 (58.3%)	0.540	OR = 0.78 (0.33–1.87)
Cesarean delivery, *n* (%)	41 (75.9%)	26 (72.2%)	0.700	OR = 1.22 (0.48–3.14)
Antenatal steroids, *n* (%)	50 (92.6%)	34 (94.4%)	0.750	OR = 0.75 (0.13–4.45)
PROM, *n* (%)	11 (20.4%)	5 (13.9%)	0.420	OR = 1.59 (0.50–5.01)
APGAR (5th min); median (IQR)	9.0 (7.0–9.0)	8.0 (7.0–9.0)	0.12	—
CRIB-II score; mean ± SD	7.0 ± 1.5	7.1 ± 1.6	0.780	− 0.10 (− 0.74–0.54)
Postnatal age at enrollment (h); mean ± SD	6.3 ± 2.3	6.4 ± 2.2	0.860	− 0.10 (− 1.00–0.80)
RDS grade (X-ray); median (IQR)	3 (2–3)	3 (2–3)	0.950	—
Duration of NIV (days); mean ± SD	6.57 ± 3.54	7.62 ± 3.68	0.18	− 1.05 (− 2.60–0.50)

*GA* gestational age, *SD* standard deviation, *n* number, *C.S.* cesarean section, *IQR* interquartile interval (25–75th percentiles), *p*-value for comparing the studied groups, *CPAP* continuous positive airway pressure, *NIPPV* noninvasive positive pressure ventilation, *NHFOV* nasal high frequency oscillatory ventilation, *PROM* premature rupture of membrane, *CRIB-II* Clinical Risk Index for Babies II, *RDS* respiratory distress syndrome, *OR* odds ratio, *CI* confidence interval, *MD* mean difference

Regarding diaphragmatic ultrasound parameters, mean DTF values did not differ significantly among CPAP, NIPPV, and NHFOV groups at the first or the second measurements (*p* = 0.134 and 0.319, respectively). For DE, first measurement values were significantly higher in the NHFOV group compared with CPAP (*p* = 0.034), while no significant differences were found between other groups. By the second measurement, DE values showed no significant differences across all three groups (*p* = 0.197) (Table 4).

**Table 4:** Diaphragmatic ultrasound parameters (DTF and DE) and TFC (absolute and indexed) among the studied groups

		CPAP; (*n* = 30)	NIPPV; (*n* = 30)	NHFOV; (*n* = 30)	***p***
DTF (%); mean ± SD	** 1 st measurement**	31.77 ± 7.55	29.50 ± 7.92	34.10 ± 10.58	0.134
	**2nd measurement**	31.77 ± 10.65	33.90 ± 9.95	35.57 ± 8.36	0.319
DE (mm); mean ± SD	** 1 st measurement**	2.74 ± 0.54	2.93 ± 0.61	3.12 ± 0.61	0.045^*^
		*p*_1_ = 0.430, ***p***_**2**_ = 0.034^*^, *p*_3_ = 0.405			
	**2nd measurement**	2.73 ± 0.73	2.98 ± 0.57	3.0 ± 0.62	0.197
TFC (absolute, 1/kΩ); mean ± SD	** 1 st measurement**	27.50 ± 5.71	29.17 ± 5.07	27.33 ± 6.98	0.228
	**2nd measurement**	35.48 ± 4.56	29.83 ± 5.08	29.0 ± 6.98	< 0.001^*^
		*p*1 = 0.001^*^, *p*2 < 0.001^*^, *p*3 = 0.600			
TEF (indexed, ml/kg); mean ± SD	** 1 st measurement**	18.10 ± 3.74	19.60 ± 3.41	18.10 ± 4.62	0.294
	**2nd measurement**	23.30 ± 3.00	20.00 ± 3.41	19.20 ± 4.62	< 0.001*
		*p*1 = 0.001*, *p*2 < 0.001*, *p*3 = 0.449			

*DE* diaphragmatic excursion, *DTF* diaphragm thickening fraction, *TFC* thoracic fluid content, *SD* standard deviation, *p*-value for comparing the studied groups, *p*1 *p*-value for comparing between CPAP and NIPPV, *p*2 *p*-value for comparing between CPAP and NHFOV, *p*3 *p*-value for comparing between NIPPV and NHFOV, *CPAP* continuous positive airway pressure, *NIPPV* noninvasive positive pressure ventilation, *NHFOV* nasal high-frequency oscillatory ventilation

^*^Significant *p*-value < 0.05

Regarding absolute and indexed TFC values, they showed no significant differences across all groups (absolute *p* = 0.228; indexed *p* = 0.294) at the 1 st measurement. However, by the second measurement, CPAP neonates had significantly higher both absolute and indexed TFC values compared to NIPPV (*p* = 0.001) and NHFOV neonates (*p* < 0.001), while no significant difference was noted between NIPPV and NHFOV groups (Table 4).

Regarding diaphragmatic ultrasound and NIV outcome, across all three NIV modalities (CPAP, NIPPV, and NHFOV), both DTF and DE were significantly higher in neonates who showed successful NIV compared with those who failed at both the initial and the second measurements (all *p* < 0.05) (Table 5).

**Table 5:** Comparison between successful and failed cases for each study group regarding DTF, DE, and TFC

		CPAP success; (*n* = 15)	CPAP failure; (*n* = 15)	*p*	MD; (95% CI)
DTF (%); mean ± SD	** 1 st measurement**	35.27 ± 5.13	28.27 ± 8.08	0.008^*^	
	**2nd measurement**	39.0 ± 4.75	24.53 ± 10.01	< 0.001^*^	+ 14.47 (8.56–20.38)
DE (mm); mean ± SD	** 1 st measurement**	3.09 ± 0.32	2.39 ± 0.50	< 0.001^*^	
	**2nd measurement**	3.27 ± 0.38	2.19 ± 0.58	< 0.001^*^	+ 1.08 (0.73–1.43)
TFC (absolute, 1/kΩ)'; mean ± SD	** 1 st measurement**	27.63 ± 6.33	27.25 ± 5.51	0.840	
	**2nd measurement**	35.68 ± 4.30	35.31 ± 4.95	0.780	+ 0.37 (− 2.74–3.48)
TEF (indexed, ml/kg); mean ± SD	** 1 st measurement**	18.21 ± 4.20	17.90 ± 3.60	0.839	
	**2nd measurement**	23.50 ± 2.80	23.22 ± 3.30	0.778	
		**NIPPV success (*n* = 19)**	**NIPPV failure (*n* = 11)**	***p***	
DTF (%); mean ± SD	** 1 st measurement**	33.63 ± 4.45	22.36 ± 7.61	< 0.001^*^	
	**2nd measurement**	39.74 ± 4.77	23.82 ± 8.35	< 0.001^*^	+ 15.92 (10.49–21.35)
DE (mm); mean ± SD	** 1 st measurement**	3.27 ± 0.39	2.33 ± 0.44	< 0.001^*^	
	**2nd measurement**	3.29 ± 0.32	2.45 ± 0.52	< 0.001^*^	+ 0.84 (0.52–1.16)
TFC (absolute, 1/kΩ); mean ± SD	** 1 st measurement**	26.67 ± 3.79	33.24 ± 4.41	< 0.001*	
	**2nd measurement**	27.06 ± 4.15	34.68 ± 3.39	< 0.001*	− 7.62 (− 10.53 to − 4.71)
TEF (indexed, ml/kg); mean ± SD	** 1 st measurement**	17.89 ± 2.50	22.32 ± 3.01	< 0.001*	
	**2nd measurement**	18.22 ± 2.83	23.30 ± 2.30	< 0.001*	
		**NHFOV success (*n* = 20)**	**NHFOV failure (*n* = 10)**	***p***	
DTF (%); mean ± SD	** 1 st measurement**	37.80 ± 7.22	26.70 ± 12.62	0.024^*^	
	**2nd measurement**	39.0 ± 5.72	28.70 ± 8.81	0.001^*^	+ 10.30 (5.45–15.15)
DE (mm); mean ± SD	** 1 st measurement**	3.48 ± 0.29	2.41 ± 0.42	< 0.001^*^	
	**2nd measurement**	3.31 ± 0.43	2.39 ± 0.47	< 0.001^*^	+ 0.92 (0.55–1.29)
TFC (absolute, 1/kΩ); mean ± SD	** 1 st measurement**	23.07 ± 2.19	35.67 ± 5.53	< 0.001*	
	**2nd measurement**	24.45 ± 2.55	38.33 ± 5.41	< 0.001*	− 13.88 (− 16.91 to − 10.85)
TEF (indexed, ml/kg); mean ± SD	** 1 st measurement**	15.34 ± 1.49	23.62 ± 3.74	< 0.001*	
	**2nd measurement**	16.19 ± 1.70	25.38 ± 3.59	< 0.001*	

*DE* diaphragmatic excursion, *DTF* diaphragm thickening fraction, *TFC* thoracic fluid content, *SD* standard deviation, *CPAP* continuous positive airway pressure, *NIPPV* noninvasive positive pressure ventilation, *NHFOV* nasal high-frequency oscillatory ventilation, *OR* odds ratio, *MD* mean difference, *C.I.* confidence interval

^*^Significant *p*-value < 0.05

To address potential confounding variables, multivariable logistic regression analyses were performed, including GA, BW, either DE, or DTF as predictors of NIV failure. In the model including DE, lower DE independently predicted NIV failure (adjusted OR = 0.38, 95% CI 0.20–0.73, *p* = 0.004), while GA and BW were not significant (*p* = 0.42 and 0.45, respectively. In the model incorporating DTF, DTF also remained an independent predictor of NIV failure (adjusted OR = 0.89, 95% CI 0.83–0.96, *p* = 0.003), independent of GA (*p* = 0.47) and BW (*p* = 0.49) (see Online Resource 1).

When stratified by NIV outcome, TFC values (absolute and indexed) were significantly higher in failed cases compared with successful ones in the NIPPV and NHFOV groups, at the first and second measurements (all *p* < 0.001). In contrast, no significant difference in TFC was observed between CPAP successes and failures (Table 5).

Additionally, diaphragmatic indices at the 2nd measurement (since that is the most predictive of NIV failure) showed the strongest predictive value for NIV outcomes. DTF exhibited the largest effect sizes, with mean differences (MD) exceeding 10–15% between success and failure across all modalities. DE also differed significantly, with the MD of approximately 1 mm. Conversely, absolute TFC (2nd measurement) showed minimal differences in CPAP and more uncertain but significant reductions in NIPPV and NHFOV (MD ranged from –7 to –14 units) (Table 5).

**Fig. 2:**
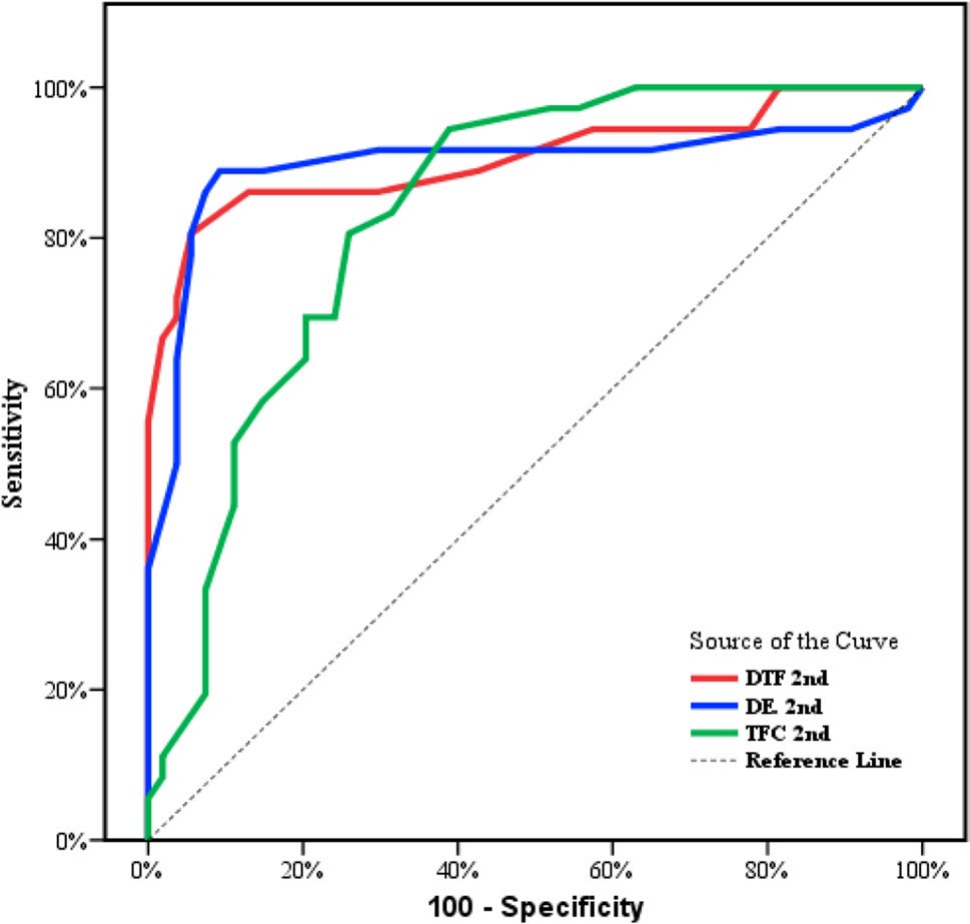
ROC curves for DTF, DE, and TEF in predicting NIV failure

ROC curve analysis was used to assess the diagnostic performance of diaphragmatic ultrasound parameters (DTF and DE) and TFC in predicting NIV failure. DTF has the highest validity with 90.6% followed by DE with 89.8% then TFC with 83.3% (Fig. 2).

Pairwise AUC comparisons using DeLong’s test confirmed that the predictive performance of DTF and DE was significantly higher than that of TFC (*p* < 0.01 for both), while the difference between DTF and DE was not statistically significant (*p* = 0.18).

In addition, when DTF and TFC were entered into a multivariable logistic regression model, both remained independent predictors of NIV failure. However, the combined model demonstrated superior discriminative performance compared with either parameter alone, yielding an AUC of 0.93 (95% CI 0.89–0.97), versus AUC 0.89 (95% CI 0.83–0.94) for DTF and AUC 0.81 (95% CI 0.73–0.88) for TFC. The DeLong pairwise comparison confirmed that the combined model significantly outperformed TFC alone (*p* < 0.001) and, though not significantly, improved over DTF alone (*p* = 0.07).

**Fig. 3:**
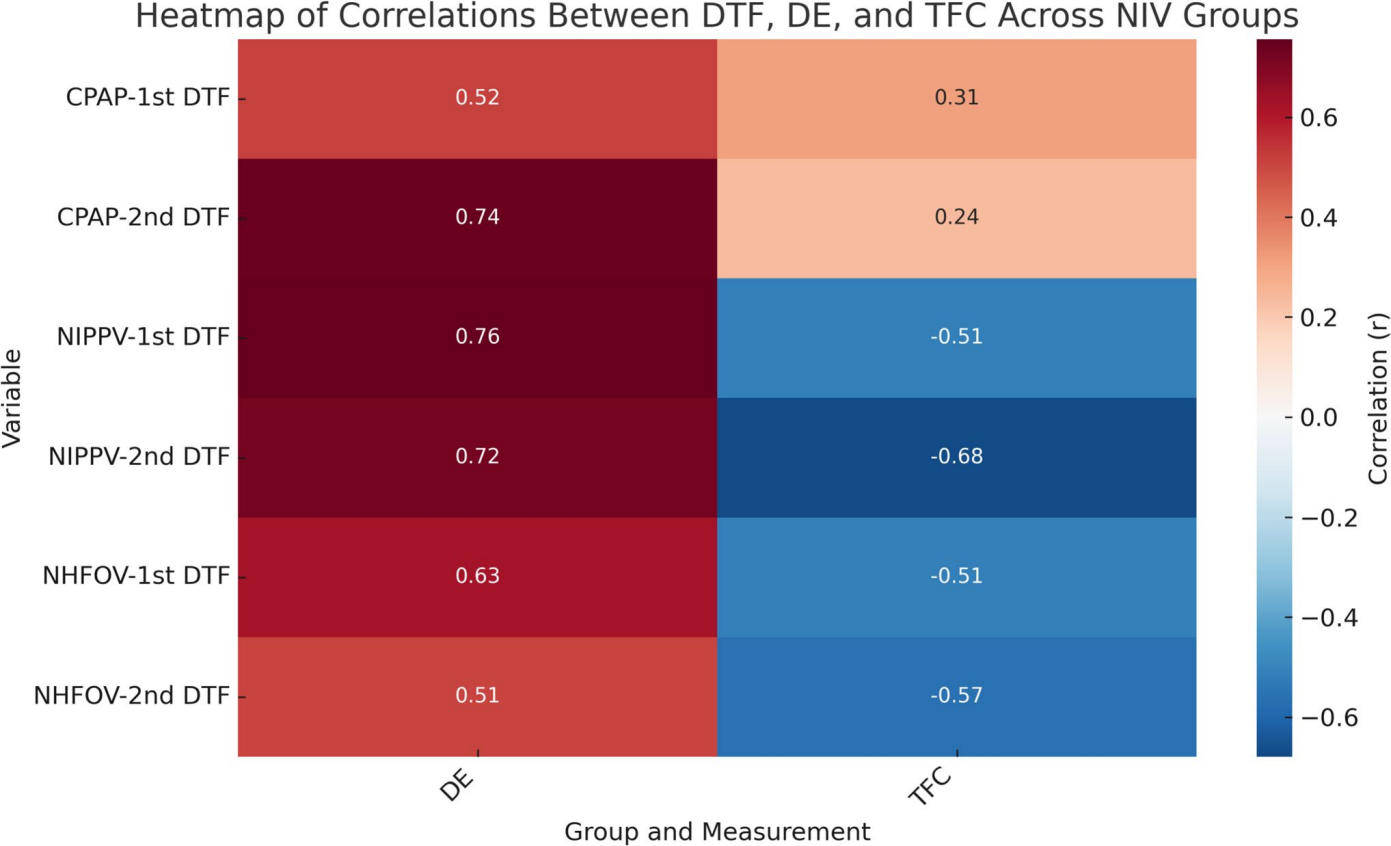
Heatmap of correlations illustrating the strength and direction of correlations between DTF, DE, and TFC across CPAP, NIPPV, and NHFOV groups. Positive correlations are shown in red and negative correlations in blue, with the color intensity reflecting correlation strength (*r* values)

Regarding the correlation between diaphragmatic parameters and TFC, DTF correlated positively and significantly with DE in all three groups (*r* ≈ 0.50 to 0.75, *p* < 0.01). In contrast, DTF had a significant negative correlation with TFC in the NIPPV and NHFOV groups (*r* ≈ − 0.5 to − 0.7, *p* < 0.01) with no significant correlation regarding cases of the CPAP group (Fig. 3).

## Discussion

To the best of our knowledge, this is the first prospective randomized controlled study to evaluate and compare diaphragmatic ultrasound parameters (DTF and DE) across three NIV modalities (NCPAP, NIPPV, and NHFOV) used as primary respiratory support in preterm neonates with RDS.

The current study highlights the utility of diaphragmatic ultrasound as a reliable predictor of NIV outcomes in preterm neonates with RDS.

At the group level, DTF values did not show significant differences among CPAP, NIPPV, and NHFOV cases, whereas DE was significantly higher in the NHFOV group compared with the CPAP group at the first measurement. While NHFOV ensures more effective alveolar ventilation and gas exchange than CPAP or NIPPV modalities [16], it may better preserve diaphragmatic performance.

On the other hand, when neonates were classified based on NIV outcome (success vs. failure), both DTF and DE were consistently higher in infants with successful NIV compared to those who showed NIV failure, irrespective of the NIV mode.

The lower DTF and DE observed in neonates who failed NIV may be due to both impaired diaphragmatic performance and developmental immaturity. The diaphragm in preterm infants, particularly those < 32 weeks’ gestation, has a lower proportion of fatigue-resistant type I fibers and reduced oxidative capacity, predisposing them to early exhaustion under increased respiratory load, as shown in previous research that found a positive association between diaphragmatic indices and maturity [20, 21]. Additionally, in patients with RDS or transient tachypnea of neonates (TTN), diaphragmatic function may differ between groups due to variations in chest wall compliance or lung tissue stiffness [22].

However, in this study, GA and BW showed no significant difference between the success and failure groups. In addition, DE/DTF independently predicted NIV failure after adjustment for GA and BW. Therefore, the current study suggests that the reduction in DTF and DE among failed cases is more likely due to functional fatigue and early diaphragmatic exhaustion, rather than immaturity, secondary to the elevated work of breathing. Such functional deterioration could limit the neonate’s ability to maintain effective spontaneous respiratory effort, ultimately precipitating NIV failure.

These findings highlight that diaphragmatic indices (DTF and DE) could serve as early and dynamic indicators of respiratory muscle performance, capturing subclinical fatigue before overt clinical deterioration occurs. Consequently, incorporating diaphragmatic monitoring into NIV management protocols may help clinicians in the early detection of impending NIV failure and to optimize respiratory support strategies.

These findings are in line with those of Abdel Rahman et al. [23], who reported that both DTF and DE were significantly reduced in infants who failed weaning from mechanical ventilation compared with those who succeeded.

In contrast, Gupta et al. [24] found that increased DE and selective changes in DTF predicted CPAP failure. Unlike the current study, which consistently demonstrated reduced DTF and DE as markers of poor outcomes across the three NIV modalities, Gupta et al. assessed infants only on CPAP and performed measurements before and immediately after initiation. Differences in study design, patient population, and timing of assessments may account for this discrepancy.

ROC curve analysis further confirmed the strong predictive value of the diaphragmatic parameters. Regarding DTF, its accuracy was 0.90 at the 2nd measurement, with sensitivity and specificity both exceeding 85%. Similarly, DE demonstrated excellent predictive value, with an accuracy of 0.89. These values validate DTF and DE as highly sensitive and specific indicators of NIV outcome.

TFC, assessed by EC, reflects both intravascular and extravascular thoracic fluid status, and previous studies demonstrated strong correlations between TFC and ultrasound estimates of extravascular lung water [13]. Thus, elevated TFC values may indirectly indicate the degree of lung congestion, a known risk factor for respiratory support failure and difficulty in weaning.

This study demonstrated that TFC was significantly higher in neonates who failed NIV in the NIPPV and NHFOV groups, while no significant difference was observed in the CPAP group. This pattern suggests that fluid overload has a greater impact on outcomes in infants requiring more advanced NIV (NIPPV and NHFOV) but is less significant in milder classic RDS cases managed with CPAP.

Additionally, TFC is known to vary by primary diagnosis, disease pathophysiology, and severity; it is typically higher in TTN [25], but not highly elevated in classic RDS cases requiring surfactant [26]. These data provide a physiological rationale for our subgroup results as neonates with higher early interstitial fluid would be expected to show elevated TFC requiring higher NIV support and a greater risk of NIV failure, whereas other classic RDS cases fail primarily due to impaired diaphragmatic mechanics rather than fluid load.

Accordingly, in the current study, the discriminatory value of TFC was significant in the non-CPAP modalities, while diaphragmatic indices (DTF/DE) remained strong predictors across all modes. In addition, pairwise AUC comparisons using DeLong’s test confirmed that the predictive performance of DTF and DE was significantly higher than that of TFC (*p* < 0.01 for both).

These findings support using TFC as a complementary tool alongside diaphragmatic ultrasound, with interpretation anchored to the underlying diagnostic phenotype and disease severity. This was highlighted by the improved predictive performance of combining DTF and TFC for NIV failure, achieving an AUC of 0.93, which indicates excellent discrimination.

The correlation analysis, conducted in this study, supports the presence of a clear relationship between diaphragmatic function and fluid burden. Reduced DTF correlated with higher TFC in the NIPPV and NHFOV groups, underscoring the physiological interaction between pulmonary congestion and diaphragmatic performance, as fluid overload may impair lung mechanics and diaphragmatic performance.

This study also revealed a positive correlation between DE and DTF across all study groups reflecting their shared physiological basis in diaphragmatic contraction while each provides unique functional information (DE indicates the diaphragm’s positional change during breathing, reflecting lung expansion and correlating positively with tidal volume, and DTF measures the thickening of diaphragmatic muscle fibers during contraction, reflecting the diaphragm’s intrinsic contractile capability) [27], which improves the accuracy of ultrasonographic evaluation. This interrelationship supports the integration of both DE and DTF as reliable markers in evaluating diaphragmatic performance and predicting NIV outcomes.

This observed correlation matched the results of Gadwal et al. [28]’s study, which found a statistically significant positive correlation between DE before extubation and each of the values of DTF before and after extubation. Furthermore, there were significant positive correlations between DE after extubation and each of the values of DTF before and after extubation, underscoring a dynamic interaction between diaphragmatic function and extubation readiness.

Although NIV success rates were not statistically different, NHFOV was associated with a significantly shorter duration of support compared to both CPAP and NIPPV. This finding is clinically relevant, as reduced duration of NIV may contribute to decreased complications such as nasal trauma, feeding difficulties, or prolonged oxygen requirement. Additionally, shorter NHFOV duration likely reflects its superior ability to provide more effective alveolar ventilation and gas exchange than CPAP or NIPPV, which aligns well with findings from prior studies [16].

The current study has some limitations. Firstly, it was conducted in a single tertiary center with a relatively small sample size, which may restrict the generalizability of the findings. Secondly, although randomization with allocation concealment was applied, blinding of caregivers and outcome assessors was not feasible due to the nature of the respiratory interventions, raising the potential for performance or detection bias. Additionally, the timing of the second evaluation was not standardized, as it was performed either 24 h after NIV weaning in successful cases or immediately before intubation in cases with NIV failure, which may have introduced some variability. Furthermore, diaphragmatic ultrasound is a promising tool; however, its interpretation requires adequate training and expertise, which may limit widespread applicability in routine NICU practice.

## Conclusion

Diaphragmatic ultrasound offers a simple, non-invasive, and reliable method for predicting NIV failure in preterm neonates with RDS. DTF and DE showed superior predictive accuracy compared with TFC-derived EC, and their combination (DTF–TFC model) provided the highest overall discriminative power. These findings highlight the clinical value of integrating early assessment of DTF, DE, and TFC into routine NICU practice, as these parameters may represent a valuable tool for monitoring and guiding clinical decision-making during the early application of NIV to preterm infants with RDS.

## Supplementary Information

Below is the link to the electronic supplementary material.

Supplementary file1 (PDF 201 KB)

## Data Availability

The corresponding author may provide the datasets used and/or analyzed during the present investigation as MS Excel files (.xlsx) upon reasonable request.

## Abbreviations

BPD: Bronchopulmonary dysplasia
BW: Birth weight
CPAP: Continuous positive airway pressure
DE: Diaphragmatic excursion
DTF: Diaphragmatic thickness fraction
EC: Electrical cardiometry
GA: Gestational age
IMV: Invasive mechanical ventilation
NHFOV: Nasal high frequency oscillatory ventilation
NIMV: Nasal intermittent mandatory ventilation
NIV: Non-invasive ventilation
RDS: Respiratory distress syndrome
TFC: Thoracic fluid content
TTN: Transient tachypnea of neonate
